# Hypnotism

**Published:** 1904-09

**Authors:** Charles Gilbert Chaddock


### HYPNOTISM.[1]

BY CHARLES GILBERT CHADDOCK, M.D.

Gentlemen,—In a lecture which I had the honor to give before the Senior class of this school about a year ago, I discussed the subject of "Pain,"[2] in its physiologic and psychologic relations. In that discussion I had much to say about the mental aspect of pain and the ways in which consciousness of pain could be modified by influencing the state of mind of the individual suffering it. In the cases cited as demonstrating the variability of pain with the variations of the state of consciousness, I made no direct reference to hypnotism, and attempted to show the effects of suggestion alone to induce insensibility to pain. It seems, then, appropriate to continue the discussion, to some extent, of the influence of suggestion as it is shown in the practice of hypnotism.

Hypnotism is a term derived from the word "hypnosis," which was first introduced by an English physician, Braid, in 1841. It is a word of Greek origin, which signifies sleep, and was used by Braid to cover a peculiar state of somnolence which he induced in certain individuals by means of certain procedures. Braid was the first observer who attempted to investigate scientifically certain peculiar states of mind of spontaneous or induced origin, which had previously been regarded as due to animal magnetism, but he never went so far as to deny the existence of animal magnetism.

A French charlatan, Mesmer, in the latter part of the eighteenth century, made himself famous by the wonders he performed with the aid of so-called animal magnetism, and so celebrated did his work become that his name became attached to the procedures which he employed and the state of mind which he induced. Until Braid's work was published these phenomena were classed under the term Mesmerism; thus animal magnetism, Mesmerism, Braidism, and hypnotism are terms for the same phenomenon; the name has been changed with variation of the conception of the nature of the phenomenon.

---

[1] A lecture delivered to the Senior Class of the Marion-Sims Dental College.

[2] International Dental Journal, August, 1903.

Notwithstanding the scientific spirit in which Braid approached the subject, it did not emerge from the realm of mystery until more light was thrown upon it by the studies of Charcot and the School of Nancy in the seventies. Even under the search-light of a scientific mind like that of Charcot, the mysterious subject of hypnotic phenomena was only elucidated after years of study and observation, and even at the present time there are striking differences of opinion between rival observers; which only serves to emphasize the difficulties the human mind has to encounter in forming just opinions about the phenomena of life.

It would be unjust to omit reference to an earlier observer who clearly explained the nature of hypnotic facts. In 1784 the subject of animal magnetism was brought before the French Academy of Sciences for discussion at the time when Mesmerism was at its zenith of popular interest. Many were the occult and mysterious explanations offered for these remarkable manifestations. In reference to this animated search for supernatural causes, an extract from a report of Bailly before the French Academy of Sciences is worthy of citation: "In searching for an imaginary cause for animal magnetism the actual power that man exercises over his fellow-beings without the immediate and evident intervention of a physical agent, is recognized." By this you will see it is meant that the mental state of one man is influenced by the mental state of another or others, and it is a fact that it is not necessary to appeal to the supernatural to explain it. Bailly in support of his assertion had shown "that the power of man over the imagination can be elaborated to an art, at least in relation to such persons as believe in the possibility of such things." In this statement Bailly explains perfectly the phenomena of Mesmerism, which was, in fact, only the art of suggestion, exercised upon those who were by belief or faith or prejudice in awe before the supernatural.

Mesmerism, Braidism, and hypnotism are not new. The phenomena to which these terms have been successively applied are as old as humanity itself. You all know that for centuries the fakirs of India have practised upon the credulity of a fanatical and ignorant people by means which to-day we speak of as suggestion; and we have no farther to go than to our immediate neighbors to realize the potency of the wonder-worker. The travelling quack, the painless tooth-puller of the country district, is practising in our day what the fakir of India has practised for ages.

In discussing a subject it is necessary to define, if possible, its limitations; in other words, we must attempt to understand what is meant by hypnotism, hypnosis, hypnotic suggestion, etc.

Hypnotism is a general term applied to all phenomena which characterize the state of hypnosis; therefore it will be understood if a satisfactory limitation can be given to the term hypnosis. Hypnosis is a peculiar state of consciousness which manifests itself under certain circumstances, characterized by a limitation of ideas to a narrow circle within which the subject acts and out of which he cannot pass without the aid of extraneous ideas or influences which have no immediate relation to the series or circle of ideas which dominate consciousness. It should be added to justify the term hypnosis that the individual manifests during the time of limitation of consciousness a certain state of sleep or somnolence but remains more or less subject to the influence of others.

The first observer who clearly stated the theory of suggestion, as it is understood to-day, was Liebeault, in 1866. He refers all phenomena of animal magnetism, so-called, to the influence of suggestion reaching the subject through physiologic avenues. His views became the starting-point of the School of Nancy, which maintains that the phenomena of hypnosis are normal, and claims that eighty per cent. of human beings can be hypnotized.

In contradiction to the Nancy School arose that of the Salpétrière or the Charcot School, which claimed that the phenomena of hypnotism were abnormal and presented certain somatic or bodily symptoms which were characteristic and pathognomonic. While the Nancy School regarded all the manifestations of the hypnotized subject to be due to the effect of idea, the Charcot School maintained that certain physical means exercised a direct influence upon the patient without the intervention of the mind.

There flourished at the same period a belief in the power of medicines to exercise their effects at a distance. These views were maintained until his death by Luys, notwithstanding the scientific demonstration that his ideas were entirely false and that the phenomena he attributed to the influence of medicine were the result exclusively of suggestion.

At the present time the contradictions between the School of Nancy and the Charcot School continue to exist, though some of the early elements of dispute have been eliminated, and the question has now become narrowed to the discussion as to whether hypnosis and hypnotic manifestations form a part of the disease known as hysteria, or are without pathologic significance.

In my lecture of last year, I pointed out the efficacy of suggestion as a means of overcoming pain, and showed how almost universally human beings are open to suggestion and persuasion; how our habitual beliefs and our manner of thinking are the result, in a large measure, of suggestion.

The efficacy of an idea given to another depends largely upon the mental training and habits and beliefs of the individual, as well as upon the nature and source of the idea which is intended to influence him; thus there must be great variability in the susceptibility of individuals to suggestion, though all probably are more or less subject to its influence. Probably the extravagant claims made for hypnotism by the Nancy School, in the belief that the vast majority of human beings can be hypnotized, is based upon this general fact of almost universal suggestibility; but it is a long step from mere suggestibility to true hypnosis, and in reality the persons in whom a true hypnosis can be induced make up but a small portion of humanity in general.

Before attempting to explain the nature of hypnosis, and before making any statement of its uses or abuses and possible injurious effects, it will be well to give you some idea of the means by which the condition may be induced in certain susceptible persons.

In the practice of Mesmerism, so-called, sleep is induced by the belief that some supernatural indefinable influence (fluid) leaves the operator and effects the subject in such a way as to induce in him the conditions which have been predicted; for example, the operator makes certain passes in the neighborhood of the subject, all the time stating that the result will be sleep, and that in this state he will become subject to the will of the operator, performing all that he is commanded to perform and with inability to resist the commands. In order to place a subject in a receptive state of mind, certain procedures are also employed, such as fixing with the eyes a bright object, with the command that the subject is not to wink and that sleep will follow. The natural result of fixing the eyes on a given object is a burning of the eye-lids that induces heaviness of the eyes, which naturally suggests sleep, even though the idea of sleep has not been otherwise imparted to the subject. In this way susceptible persons pass into an hypnotic state. Braid was accustomed to use such means with verbal suggestion, though he believed that fixation was an essential element. Later, as has already been explained, Liebeault discovered the essential element in the idea imparted, and that all that was essential was verbal suggestion. The method now generally employed is to place the subject in a position of repose and suggest that the eyes are heavy and that drowsiness is creeping over the individual. Certain external circumstances lend weight to these ideas. It is especially effectual to place the subject to be operated upon in the presence of hypnotized or sleeping persons for the aid lent by subconscious suggestion. If a subject be influenced, he apparently sleeps, but is in relation with the operator, and may be induced to act in accordance with ideas communicated to him by the operator. In this condition, however, the subject is rarely, if ever, *absolutely* in the power of the operator. There is a certain limit beyond which suggestions have no power to influence the individual. Thus it is very easy to induce the hypnotized person to perform silly acts or those which are without special moral meaning, or which are not in opposition to his moral training; but even in the most susceptible hysteric patients it is practically impossible experimentally to induce them to attempt acts which are manifestly criminal, providing that such acts are out of harmony with their training and habitual attitude of mind. However, it is true that in the hypnotic state, the subject accepts ideas more readily than in the waking state, and on account of this, during hypnosis, ideas may be imparted to the subject which overcome ideas that are habitually active during the moral waking state. This makes it possible to remove or overcome certain mental or bodily symptoms which are dependent upon idea. Thus psychic pain may be removed by suggestion, and paralysis, anæsthesia, contracture, etc., that have as their basis an idea, conscious or subconscious, frequently may be overcome and permanently cured by suggestion in the hypnotic state. On the other hand, by the same means (suggestion) similar phenomena may be made to appear in the hypnotic state; so that after waking from the hypnotic state the subject presents pain, paralysis, tremor, contracture, anæsthesia, etc.

The hypnotic state of consciousness is peculiar in this, that it is a secondary state, or a state differing from that of moral consciousness, so that the person that has been repeatedly hypnotized comes to have a double consciousness in a sense. During hypnosis memory of the events of the normal waking state is quite obliterated; and on the contrary, in the normal waking state memory of the events during the continuance of the hypnosis is wanting, but with repetition of hypnosis there is memory for the facts of previous hypnotic states. In certain individuals this doubling of consciousness is so marked, and the two states are so distinct, that actually two personalities are created.

Since many of the writings on hypnosis still refer to the original conception of Charcot, it may be well to review the phenomena which were regarded by that observer as essential in so-called "Grand Hypnotism."

It should be stated at once that grand hypnotism as understood by Charcot was observed only in persons subject to hysteria in its most marked form. The procedure employed in general was to suggest to the patient that she would fall asleep, at the same time the operator pressed with his fingers on the eyeballs after the patient had closed the eyes. The patient passed into a somnolent state which lasted indefinitely, and in which she was not open to verbal suggestion. Owing to the apparent state of relaxation, with resemblance to sleep, this condition was called lethargy. Without interference from the operator this state would last indefinitely. In the state of lethargy, however, certain procedures produced peculiar phenomena. If the operator stroked a limb, for example the forearm, the muscles touched would become forcibly contracted. This contracture would continue indefinitely, and remain after the patient had been wakened, if pains had not been taken to remove it by some procedure—either by forcible pressure on the muscles or by blowing lightly upon them. This phenomena of contracture could also be induced by approaching a magnet to the patient.

If now (during lethargy) the patient's eyelids were raised by the operator, they remained open and the gaze fixed. The raising of the eyelids induced a secondary altered state. In this condition the patient remained fixed in the attitude which she happened to be at the moment the eyelids were raised, but this attitude could be altered by the operator, and whatever position was given to the limbs or body was maintained indefinitely; in other words, this condition was one of catalepsy. In this cataleptic state the patient was more open to suggestion, but still seemed to be dominated by a single fixed idea, or in a state of suspension of mental activity. Mainly suggestion exercised its effect through attitude, for the facial expression changed in harmony with the attitude (prayer). The phenomena of contracture evocable in the state of lethargy could not be induced here. If the eyelids were mechanically closed the patient immediately passed again into the state of lethargy, from which she could again be changed by suggestion into an apparently normal waking state, but which is an exceptional mental condition in which she is entirely subject to the will of the operator. This condition the Charcot School called somnambulism, for the reason that the patient walked, talked, and acted without special abnormality, and accepted all possible ideas from the operator; thus a hypnotized patient in a condition of somnambulism may be made to believe that she hears or sees anything which the operator suggests, and many of the bodily functions may be influenced in the same way; thus all the symptoms of hysteria may be brought into being by suggestion, such as anæsthesia, paralysis, contracture, and attacks of grand hysteria (convulsions).

Charcot's study of these three states was at fault in that he failed to recognize that all three states were equally the result of suggestion; for he believed that they were characteristic of hypnosis, and that stroking, pressing, blowing, and magnets exercised a direct physical effect to induce the changes observed. However, he admitted that there were certain cases in which these phenomena did not occur, or could not be induced in their fully developed form. The similarity between lethargy, catalepsy, and somnambulism, as manifested in his patients, and certain phenomena characteristic of hysteric persons, led at once to the view that these conditions were identical; in other words, that grand hypnosis and grand hysteria were the same thing.

Out of the error which led the School of Charcot to regard certain phenomena of hypnotism to be due to the specific physical effect of certain procedures on hypnotized subjects, and the probable truth that the characteristic conditions of hypnosis are identical with those observed in certain persons subject to hysteria, resulted the controversy which has waged for years between that school and the adherents of the School of Nancy. On the one hand, the influence of suggestion was in part overlooked; on the other, namely, by the adherents of the School of Nancy, the identity of hysteria and hypnosis has been entirely ignored. This controversy has been waged especially around the therapeutic value of hypnotism. The School of Charcot has from the first been very sceptical as to the therapeutic value of hypnotism in the treatment of disease, whereas the School of Nancy has gone to great lengths in its advocacy of suggestion and hypnotism as means of cure of all forms of disease. The Parisian school (Charcot) has at no time underestimated the value of *waking suggestion* as a possible means to cure all disease of psychic origin, and in this respect it would seem to an unprejudiced observer that the diversity of views between the two schools under consideration were one of name rather than of nature; in fact, the School of Nancy has gradually abandoned the use of hypnotic suggestion *per se* and advocates the use of suggestion given in the waking state merely, claiming that results can be obtained which compare favorably with those which were formerly said to be possible only as a result of suggestion given during hypnosis. To-day the adherents of the School of Charcot hold practically the same view with regard to the therapeutic uses of suggestion, but they maintain the view that hypnosis, which in itself represents only the highest degree of suggestibility, is only a manifestation of hysteria, and can be induced only in those subject to hysteria; and further, that all the cures that have been reported as due to hypnotic suggestion are due to the fact that the symptoms or conditions that have been cured or changed by hypnotic suggestion are hysteric in nature.

Probably much of this difference of opinion arises from lack of identity of understanding of what is meant by hysteria.

It would take me too far to go into detail regarding the pathology of this mental disease, but it may be stated that the phenomena of grand hysteria and grand hypnosis are practically the same; in the one case the symptoms arise spontaneously; in the other they are artificially or secondarily induced.

What are the uses of suggestion in general and the uses of hypnotic suggestion in particular?

Waking suggestion, as we see in every-day life, exercises a profound influence upon the mental state of individuals, and is perhaps the most important aid to the physician in his treatment of patients, whether they are suffering with physical ailments or those due to idea. To the patient suffering with actual physical disease, it lends hope and faith, and thus enhances or favors the action of drugs that have been administered with the view to cure physical disease. Oftentimes it assists the patient through a crisis, thus giving time for material remedies to act. Such cases are not instances of hysteria in any true sense of that term.

Hypnotic suggestion can only be employed in relation to conditions that are actually hysteric; in other words, in conditions that are the result of idea—psychic. By means of hypnotism or suggestion given in hypnosis, it is possible to overcome accidents or symptoms which have not yielded to waking suggestion; in other words, this procedure is only practicable with hysteric persons; and the fact that hypnosis in a strict sense can be induced is practically a demonstration of the hysteric condition of the individual. However, not all hysteric individuals can be hypnotized, and therefore the range of application of hypnotism is necessarily limited; furthermore, hysteric patients presenting hysteric symptoms of the gravest nature, even though it be possible to hypnotize them, cannot always be improved or cured. In many cases of this kind the symptoms can be removed during the continuance of the hypnotic state, but they return and persist after awakening from hypnosis.

The wide range of application of hypnotism, suggested by the writings of the School of Nancy, does not find support in the facts of experience; for to obtain results by this means it must be applied to hysteric persons, and only a certain portion of hysteric individuals can be benefited by it.

In addition to this limitation, there must be mentioned the possible dangers which attend the indiscriminate use of hypnotism. Since those in whom hypnosis can be induced are essentially hysteric, there is the danger of grave hysteric accidents or conditions arising during or as a result of hypnosis. In particular, a person predisposed may be so affected that she become thereafter subject to periodic attacks of grand hysteria, which remain refractory to all means of treatment. It has also been observed that persons frequently subjected to hypnosis become more and more easily influenced by others, until finally they become subject to auto- or self-suggestion, and thus an original condition of hysteria has been intensified or a latent hysteric predisposition has been developed into fully developed grand hysteria.

Popular and public exhibitions of wonders of hypnotism are to be most emphatically condemned; indeed such theatrical travesties should be prohibited by law. In the first place, the hypnotist who practises the art for the amusement and mystification of the public is but rarely acquainted with the nature of the instrument he wields, and in nowise responsible for the lasting harm he may cause. But the ignorant hypnotist who plays upon the credulity of the people is no more to be condemned than the ignorant newspaper writer or reporter, and the more cultivated writer of modern novels, who employs the mysteries of hypnotism to heighten interest in his impossible. I have no doubt that many educated persons entertain ideas of hypnotism gathered solely from reading such popular novels as "Trilby" and the supernatural effusions of Marie Corelli. Such popular and unscientific presentations of a subject which at first sight seems so mysterious only serve to foster in the minds of the masses a belief in the supernatural, with all the errors and mental aberations which spring from ignorance. Whether it be in the uncultured or in the educated, false ideas concerning the nature of Mesmerism and hypnotism are in a measure responsible for many prevalent errors that afflict the world to-day. The hypnotism of the Indian fakir, which can be traced back to Persia in the person of the celebrated Zoroaster, is doubtless in large part responsible for the occultism that reigns and has reigned for centuries among the Hindoos, and which has found acceptance among some of the brightest, though misguided, minds of the Occident.

Spiritualism, as it is understood to-day, was born in hypnosis (autosuggestion) and developed through suggestion.

Christian Science is perhaps the most remarkable monument ever erected by the human mind to celebrate the powers of autosuggestion. It goes without saying that were the true nature of the means by which the very remarkable results of practical Christian Science understood by its adherents, such results could not in the nature of things be obtained. All depends upon faith in the supernatural nature of the means which bring about the end. If these remarkable and sometimes exceptional results had not a reverse, it would not seem absolutely necessary to attempt to destroy the delusion of which they are the consequence. Unfortunately, the devotee of an idea or theory that is believed to be of universal application must come in conflict with actual conditions and physical obstacles. Thus great harm results. The Christian Scientist, in his ignorance of the natural limits of his "science," applies his theory to physical disease, and thus throughout our country many innocent lives are sacrificed yearly to blind and unreasoning bigotry.

When one walks the streets to-day or glances over the advertising pages of the daily newspapers, he is almost forced to the conviction that the superstition and ignorance of the Middle Ages have come down to us unchanged. Fortune-tellers, clairvoyants, mind-readers, and palmists seem to have their signs in every available window. The advertising hypnotist has now developed the school of hypnotism, which does not confine its instruction to the student in attendance, but teaches the art of hypnotism by letter.

In the same category are to be reckoned certain writers on so-called mental science, who very plausibly present the thesis, convincing to the mind of one unschooled in physiology, of the actuality of mind-reading, thought transference, and mental suggestion.

I make these cursory allusions with a view merely to point out the dangers which attend the spread of errors concerning the mind and its nature; and especially because I consider that the erroneous views still prevalent with regard to hypnotism are in some measure responsible for the facility with which these errors are accepted and propagated.

In conclusion permit me to emphasize one fact which I wish you to retain. Hypnotism is a natural, though abnormal, phenomenon. It does not depend upon supernatural influences. All the symptoms and conditions of mind which characterize the hypnotic state are subjective; that is, they belong exclusively to the individual presenting it, and its fundamental cause lies not in the influence of a second person, but in the peculiar state of the person presenting the phenomenon. If a second person can excite in a susceptible subject the phenomenon of hypnotism, it is only by means of the communication of ideas to that person by actual physical means, exactly like those we employ daily in our communication one with another.

